# Dynamic changes to the intestinal environment occur throughout recovery from experimental ischaemic stroke

**DOI:** 10.1177/0271678X251405669

**Published:** 2026-01-17

**Authors:** Rachel M L Martin, Isobel C Mouat, Robert J Whelan, Lizi M Hegarty, Lizzie F R Blunt, Christopher J Anderson, David H Dockrell, Calum C Bain, Gwo-Tzer Ho, Laura McCulloch

**Affiliations:** 1 Institute for Regeneration and Repair, Centre for Inflammation Research, University of Edinburgh, Edinburgh, UK; 2 School of Infection & Immunity, Centre for Immunobiology, University of Glasgow, Glasgow, UK

**Keywords:** Stroke, intestine, microbiota, antibodies, B cells

## Abstract

Stroke survivors can experience a plethora of complications throughout their recovery that impair quality of life and impact neurological outcomes. Intestinal dysfunction is reported to occur rapidly following stroke in both humans and animal models and alterations such as reduced barrier integrity, lymphocyte loss and an altered microbiota have been suggested to contribute to increased brain injury. Despite the persistence of gastrointestinal symptoms in many stroke survivors, how the intestinal environment changes over the course of stroke recovery remains poorly understood. Here, we use an experimental model of ischaemic stroke to determine if gastrointestinal dysfunction persists into chronic recovery. We have shown that experimental stroke leads to structural alterations to the colon, a thinned mucus barrier, impaired transit times and an altered bacterial community composition. While barrier function and transit times recover within 2 weeks, structural and bacterial community alterations remain up to 3 months after stroke and are accompanied by altered luminal antibody profiles. Together, this suggests that the gastrointestinal system is dynamically altered over the course of experimental stroke recovery and highlight intestinal dysfunction as a target to improve patient outcomes beyond the acute recovery window.

## Introduction

Stroke is a leading cause of death and disability with 143 million recorded cases of stroke worldwide in 2019 and an ever increasing prevalence.^1^ Advances in hyperacute stroke care and the introduction of thrombectomy and thrombolysis for clot removal have improved survival rates. Consequently, the number of stroke survivors is predicted to double by 2035.^2^ Non-neurological complications remain an issue during recovery and can impair neurological recovery and impact quality of life.^3,4^ Gastrointestinal effects of stroke include dysphagia,^5^ constipation^6^ and intestinal bleeding.^7,8^ These complications often persist long-term and are linked to worsened clinical outcomes from stroke.

Intestinal dysfunction is well reported acutely after stroke, including gut barrier leakiness, reduced mucus production and delayed transit time.^6,9–12^ The gut bacterial community, or microbiota, is altered within 24 h of stroke in both people and experimental animal models and dysbiosis has been independently associated with worsened functional and neurological outcomes.^7,8,13–15^ Intestinal immune homeostasis is also compromised,^16,17^ with loss of Peyer’s patch and mesenteric lymph node lymphocytes^18–21^ reported. Impairment to mucus and epithelial cell barriers are thought to facilitate translocation of bacteria, therein contributing to systemic inflammation.^11,22^ Additional factors consist of reduced food consumption or nasogastric tube feeding, recumbency and medications which alter gut transit or gastric pH, all of which can affect nutritional status and intestinal motility.

The established link between intestinal changes alongside the rapidly developing brain injury has necessitated studies focussed on hyperacute changes to the intestinal microenvironment. Interventions using antibiotic removal of the intestinal microflora or supplementation with short chain fatty acids after experimental stroke highlight the detrimental impact an altered bacterial community can have on the recovering brain.^13,23^ However, gastrointestinal issues often persist throughout recovery and remain associated with risk of infection,^5^ increased mortality^7,14^ and recurrent stroke.^8,15^ Despite the chronicity of gastrointestinal complications, temporal characterisation of the functional, physiological, immunological and microbial changes to the intestine following stroke is currently lacking.

In this study, we present the first evidence of chronic intestinal dysfunction after experimental stroke. Our findings reveal persistent macroscopic changes to colon structure, despite a normalisation of barrier function and transit time. Furthermore, we observe that the intestinal microbial community composition is altered during both acute and chronic recovery, with distinct shifts occurring in each phase, indicating that the post-stroke microbiota neither regains homeostasis, nor is stable. Lastly, we find reduced antibody coating of bacteria during chronic stroke recovery, despite increased antibody availability and normalised intestinal B cell numbers. We propose that prolonged and dynamic changes to intestinal homeostasis occur after stroke and targeting intestinal health is a viable strategy to improve neurological outcomes beyond the acute recovery period.

## Materials and methods

### Experimental stroke surgery

Experimental ischaemic stroke was achieved using the middle cerebral artery occlusion (MCAO) model with a 30 min occlusion, as previously described.^24^ Sham animals experienced the same surgical procedure, however, the filament was immediately retracted upon reaching the middle cerebral artery. Surgery was performed under isofluorane anaesthesia induced at 3.5% and maintained at 1.5% with O_2_ (200 ml/min) and N_2_O (400 ml/min). Core body temperature was maintained at 37 °C ± 0.5 °C throughout the procedure with feedback controlled heating via a small animal physiological monitoring system (Harvard Aparatus) which also allowed monitoring of heart rate, respiration and blood oxygen saturation throughout the surgical procedure and animals were recovered in cages partially placed on a heated recovery mat for a minimum of 1 h. Post-operative analgesia included application of lidocaine cream to the wound and 0.1 mg/kg buprenorphine immediately after surgery and 24 h post-surgery. Mice were recovered for 5 days, 2 weeks (12–15 days) or 3 months (61–95 days) following surgery. This study is comprised of five independent experiments and details of these, along with information on experimental design and sample collection, can be found in Figure S1. Experiments were carried out under a UK Home Office Project License in accordance with the Animals (Scientific procedures) Act 1986 and Directive 2010/63/EU and were approved by the University of Edinburgh’s Animal Welfare and Ethics Review Board. The ARRIVE guidelines were consulted for experimental design, analysis and reporting^25^ and the IMPROVE guidelines were additionally used to maximise recovery after MCAO.^26^

### 16S rRNA sequencing

To examine the composition of the bacterial microbiota, 16S rRNA sequencing was performed. DNA was extracted from 40 to 200 mg stool by the NU-OMICS DNA Sequencing facility (Northumbria, UK). Bacterial DNA was isolated with the QIAGEN PowerLyzer PowerSoil kit (Qiagen) following manufacturer’s instructions. Library preparation was carried out by NU-OMICS (Northumbria University) based on the Schloss wet-lab MiSeq SOP.^27^ Briefly, PCR was performed using the 1× KAPA2G Robust HotStart ReadyMix, 0.5 µM each primer and 1 µl of template DNA with the following cycling conditions: 95 °C 2 min, 30 cycles 95 °C 20 s, 55 °C 15 s, 72 °C 5 min with a final extension 72 °C 10 min. A negative and positive (Zymobiomics Microbial Mock community DNA standard) control sample were included in each 96-well plate and carried through to sequencing. PCR products were quantified with the Quant-iT™ PicoGreen™ dsDNA Assay (Invitrogen) and each sample was normalised to 10 nM and then pooled within each 96-well plate. Each pool was quantified using fragment size determined by BioAnalyzer (Agilent Technologies) and concentration by Qubit (Invitrogen) and pools were combined in equimolar amounts to create a single library. The library was then denatured using 0.2 N NaOH for 5 min and diluted to a final concentration of 4.5 pM, supplemented with 20% PhiX and loaded onto a MiSeq V2 500 cycle cartridge.

### 16S rRNA bioinformatics analysis

DADA2 was implemented using the Nextflow nf-core ampliseq pipeline (version 2.5) for the 16S rDNA bioinformatic analysis.^28^ Paired-end sequences were filtered and trimmed with –trunclenf 220 and –trunclenf 170 and default parameters. Taxonomy was assigned using the Silva 138.1 reference database.^29^ The phyloseq package (version 1.38.0) was used to generate a phyloseq object from the DADA2 output for downstream analysis.^30^ The amplicon sequence variant (ASV) abundance table was normalised using the total sum scaling (TSS) method to relative abundances, and low abundant ASVs were filtered out (<0.01%). Alpha diversity analyses were generated using phyloseq (version 1.38.0) and the Shannon diversity index was used. Analyses was performed on both raw unfiltered and rarefied ASV-level. Beta diversity analyses was performed with Vegan (10.32614/CRAN.package.vegan), using Bray–Curtis dissimilarity matrices from TSS normalised and arcsine square root transformed data.

### Histological analysis

Colon rolls were embedded in optimal cutting temperature compound (Tissue-Tek) and 6 μm sections were cut on an HM525 NX Cyrostat (Thermo Fisher Scientific) at −20 °C. Coronal cryosections (20 μm) were taken at 400 μm intervals throughout entirety of fresh frozen brains on a subset of animals at each stroke recovery time point. Sections were stored at −20 °C until further analysis. To assess colon morphology and quantify infarct in brains, fresh frozen tissues sections were haematoxylin and eosin (H&E) stained as previously described.^24^ To visualise the mucus barrier, cross sections of colon, with faecal content intact, were dissected into Carnoy’s fixative for 3 h before removal into 70% ethanol, processed and embedded in parrafin blocks. Sections of 6 μm were cut and deparrafinised by soaking for ten minuntes in xylene, followed by rehydration in decreasing concentrations of ethanol 100% > 95% > 80% > 70% for 5 min each. Sections were stained in haematoxylin for 5 min, washed in distilled water, stained for 30 min in Alcian Blue (pH 3.0), washed in distilled water and then stained with eosin for 3 min. Sections were then dehydrated and coverslipped as above.

### Immunofluorescence

Fresh frozen 6 μm colon sections were fixed in ice-cold acetone for 10 min, washed in 0.05% bovine serum albumin in PBS (PBS–BSA) and blocked using normal goat serum (Jackson ImmunoResearch). Primary antibodies were incubated for 1 h at room temperature or overnight at 4 °C. Tight junctions (ZO-1), epithelial cells (EpCAM) and proliferating cells (Ki-67) were detected in colon and T cells (CD3) and blood vessels (CD31) were detected in brains using the antibodies described in Table 1. Mucin producing cells were detected using the lectin Ulex Europaeus Agglutinin I (UEA I) directly conjugated to rhodamine (1:200; Vector Laboratories; RL-1062-2). Following addition of primary antibody, sections were washed in PBS-BSA and where relevant, species specific secondary antibody or streptavidin, conjugated to Alexa Fluor (AF) dyes, decribed in Table 1, were added to sections and incubated for 1 h. Sections were washed again and DAPI was added (1:5000; Thermo Fisher Scientific) for 10 min. Sections were given a final wash in PBS-BSA and mounted in Dako Fkuorescence Mounting Medium (Agilent Technologies, S3023). Control sections were incubated only with secondary antibody and/ or species specific normal serum.

**Table 1.** Primary and secondary antibodies for immunostaining.

Antigen	Conjugate	Species	Clone	Dilution	Company	Catalogue number
ZO-1	Unconjugated	Rabbit	Polyclonal	1:200	Thermo Fisher Scientific	16881365
EpCAM	Biotin	Rat	G8.8	1:500	Thermo Fisher Scientific	13-5791-80
Ki-67	Unconjugated	Rat	SolA15	1:100	Thermo Fisher Scientific	14-5698-82
CD3	Biotin	Hamster	145-2C11	1:100	Biolegend	100304
CD31	Unconjugated	Goat	Polyclonal	1:100	R&D Systems	AF3628
Rabbit IgG	Alexa Fluor 647	Goat	N/A	1:500	Thermo Fisher Scientific	A21245
Rat IgG	Alexa Fluor 555	Goat	N/A	1:200	Abcam	ab150166
Goat IgG	Alexa Fluor 488	Donkey	N/A	1:250	Thermo Fisher Scientific	A11055
Streptavidin	Alexa Fluor 488	N/A	N/A	1:500	Thermo Fisher Scientific	S32354
Streptavidin	Alexa Fluor 647	N/A	N/A	1:500	Thermo Fisher Scientific	S32357
Streptavidin	Alexa Fluor 555	N/A	N/A	1:500	Thermo Fisher Scientific	S32355

Apoptotic TUNEL^+^ cells were measured using the ApopTag Fluorescein Direct In Situ Apoptosis Detection Kit (Merck, s7110) according to the manufacturers protocol. Following TUNEL staining, immunolabelling for proliferating (Ki-67) and epithelial (EpCAM) cells was performed as described above. Sections were then washed and mounted in Dako fluorescent mounting media (Agilent Technologies, S3023).

### Image analysis

Images of colon rolls and brain sets were taken on an Axio Scan.Z1 (Zeiss). Whole tissue was scanned at 20× magnification. A single 5-day sham animal was excluded from all colon image analysis due to poor quality of tissue. Files were exported as .czi for image analysis in QuPath (version 0.4.3).

Crypt depth was measured from the base to the tip of 10 crypts, Averaged across the entirety of the colon section. Muscularis thickness was quantified similarlyat 12–18 locations along the length of the colon. Nuclear density was assessed across five regions of interest measuring 200 × 200 μm placed randomly along sections of the crypt or muscularis and determining the number of nuclei using the cell detection tool.

Ki-67, UEA-1 and ZO-1 analyses were carried out on similar regions of interest and Ki-67^+^ proliferating cells, UEA-1^+^ mucin-producing cells and ZO-1^+^ tight junctions were quantified in both the epithelial and lamina propria layers of tissue. Positive cells were detected and counted using the positive cell detection tool and normalised to the area analysed to provide UAE-1^+^ and ZO-1^+^ cells/mm^2^ for each animal.

Coronal brain sections for quantification were identified by neuroanatomical landmarks at eight specific levels.^31^ The infarcted area at each level was quantified using Zen (v3.8; Zeiss) and plotted against distance from rostral pole. Area under the curve calculations were performed in Prism (GraphPad Prism v10) to calculate infarct volume. In mice recovered 3 months after stroke, the area of the ipsi- and contra-lateral hemispheres were measured, deducting the area of ventricles where present, to give total hemisphere area. This was plotted against distance from rostral pole to calculate hemisphere volume. Immunoflurescent brain images were additionally captured on an Opera Phenix Spinning disk confocal microscope (Perkin Elmer). To quantify CD3^+^ T cells, the ipsi- and contra-lateral hemispheres were selected and positive cells detected and counted using the positive cell detection tool.

### Faecal supernatant and protein assays

Faecal pellets were suspended in PBS (100 mg faeces/1 ml of PBS). Following 20 min of incubation on ice, samples were homogenised 2 × 30 s at 6500 rpm (Precellys Evolution Touch; Bertin Technologies) and centrifuged at 8000*g* for 10 min to pellet the bacteria. The supernatant and bacterial pellet a were each snap frozen and stored at −80 °C until analysis. Total protein in faecal supernatant was measured using the Pierce BCA Protein Assay Kit (Thermo Fisher Scientific, A55865). Lipopolysaccharides (LPS) in plasma was measured with the Pierce Chromogenic Endotoxin Quant Kit (Thermo Fisher Scientific, A39553) from samples diluted 1:75 and run according to manufacturer’s instructions. Calprotectin was measured in faecal supernatant diluted 1:2 with the Mouse Calprotectin ELISA Kit (Abcam, ab263885). To measure the abundance of various cytokines and antibodies in faecal supernatant, respectively, the LEGENDplex™ MU Th Cytokine Panel (Biolegend, 741044) and LEGENDplex™ Mouse Immunoglobulin Isotyping Panel (Biolegend, 740493) kits were used. To measure the abundance of cytokines in intestinal tissue, four Peyer’s patches per animal and a 1 cm piece of colon were harvested and washed in PBS. Tissues were placed in a Precellys tube containing 1 ml RIPA lysis and extraction buffer (Thermo Fisher Scientific, 89900) with 1% Halt Protease Inhibitor Single-Use Cocktail (Thermo Fisher Scientific, 78430) and homogenised for 3 × 30 s at 6000*g*. Undiluted tissue homogenate was analysed using the LEGENDplex™ MU Th Cytokine Panel (Biolegend, 741044) acquired on an Attune NxT Flow Cytometer (Thermo Fisher Scientific). All procedures were carried out in accordance with manufacturer’s instructions.

### Gut transit time

Transit time experiments were based on previously published methods.^32^ Experiments were carried out at night under red light during the animals’ awake cycle. Mice were individually housed, fasted for 1–2 h, and orally gavaged with 200 μl FITC–dextran 70 (Sigma, 46945) prepared at 50 mg/ml in PBS. Upon gavage, access to pre-weighed normal chow was returned. Cages were examined every 3 min using UV torches and the time of the first fluorescent pellet was recorded. The amount of food eaten and faecal pellets produced was measured. Food weight data was not collected at first experimental collection and therefore data is missing for some animals (*n* = 2 sham; *n* = 3 stroke) at the 5-day timepoint.

### Isolation of intestinal leukocytes

To isolate leukocytes from the colon, tissue was cut into 1 cm pieces, washed three times with 10 ml ice cold FACS buffer (PBS with 2% FCS and 1 mM EDTA (VWR, R013)), and intra-epithelial leukocytes were isolated by incubating at 37 °C for 30 min in prewarmed stripping buffer (RPMI-1640 with 5% FCS, 1 mM DTT (Thermo Fisher Scientific, R0861) and 1 mM EDTA) while shaking at 200 rpm. Intra-epithelial leukocytes were then placed through a 100 μm sieve, washed with 10 ml ice cold FACS buffer and placed on ice until staining. To isolate lamina propria leukocytes, the remaining tissue was washed with 10 ml ice cold FACS buffer and incubated at 37 °C for 45 min in prewarmed digestion buffer (RPMI-1640 with 1 mg/ml Collagenase D (Merck, 11088858001) and 20 μg/ml DNase1 (Roche, 101104159001)) while shaking at 200 rpm; the tissue and supernatant was then passed through a 70 μm sieve and washed with 10 ml ice cold FACS buffer. Peyer’s patches were passed through a 70 μm sieve to generate single cell suspension and washed with 10 ml ice cold FACS buffer.

### Flow cytometry

Proportions of Peyer’s patch, intraepithelial and lamina propria lymphocytes were quantified by flow cytometry. Cells were incubated with Fc block (purified anti-mouse CD16/32; Biolegend, 101301), stained at 4 °C in the dark with the antibodies described in Table 2, stained with 0.1 μg/ml DAPI (4′,6-diamidino-2-phenylindole; Thermo Fisher Scientific, D3571), and analysed with an LSRFortessa II Cytometer (BD Biosciences). Single-stain controls were prepared using UltraComp eBeads (Thermo Fisher Scientific) and counting beads (123 count eBeads™ Counting Beads; Thermo Fisher Scientific). Data were analysed using FlowJo (V10).

**Table 2.** Flow cytometry antibodies.

Antigen	Fluorophore	Clone	Dilution	Company	Catalogue number
CD4	PE-Dazzle 594	RM4-5	1:200	Biolegend	100565
Ly6G	PE-Cy7	1A8	1:800	Biolegend	127618
CD19	APC	1D3	1:200	Biolegend	152410
CD19	BV650	1D3	1:200	Biolegend	115541
CD45	Alexa Fluor 700	30-F11	1:400	Biolegend	103128
CD8a	APC-Cy7	53-6.7	1:200	Biolegend	100712
CD8a	Brilliant Violet 421	53-6.7	1:50	Biolegend	100737
IgK	Brilliant Violet 711	187.1	1:20	BD Biosciences	742837
IgG	PE	Poly4053	1:100	Biolegend	405307
IgA	FITC	N418	1:100	Thermo Fisher Scientific	11-4204-82

### Antibody coating of faecal bacteria

Bacterial pellets were resuspended in 1 ml FACS buffer and filtered through 70 μm sieve. Samples were stained with 17 μM SYTO-60 (Thermo Fisher Scientific, S11342) for 30 min at 4 °C. Next, samples were stained with antibodies against IgK, IgG and IgA (Table 2) for 30 min at 4 °C and subsequently with AF488-streptavidin at 1:500 (Thermo Fisher Scientific, S32354) for 60 min at 4 °C. Data was aquired on a NovoCyte cytometer (Agilent Technologies) and data were analysed using FlowJo (V10).

### Statistical analyses

With the exception of 16S rRNA bioinformatic analysis, data presentation and statistical analyses were performed using GraphPad Prism software (version 9.5.1; GraphPad Software Inc.). For normally distributed data, differences were tested using one-way or analysis of variance (ANOVA) with Dunnett’s multiple comparisons test, comparison to naive or stroke. Data that was not normally distributed according to Shapiro–Wilk was either analysed by Kruskal-Wallis or was log transformed and analysed by one-way ANOVA, as indicated in figure legend. Data with unequal standard deviations, determined by the Brown–Forsythe test, were analysed by Brown–Forsythe and Welch ANOVA tests. Ordinal data were analysed by Kruskal-Wallis. For data with two independent variables and with different numbers of observations in each group were analysed by fitting a mixed-effects model (REML) with the Geisser–Greenhouse correction. Multivariate analysis was performed by permutational multivariate ANOVA (PERMANOVA). Results are presented as mean ± SD unless stated otherwise. *p* values indicated by asterisks as follows: ****p < 0.0001, ***p < 0.001, **p < 0.01, *p < 0.05.

## Results

### Changes to colon morphology persist across stroke recovery

**Figure 1.**
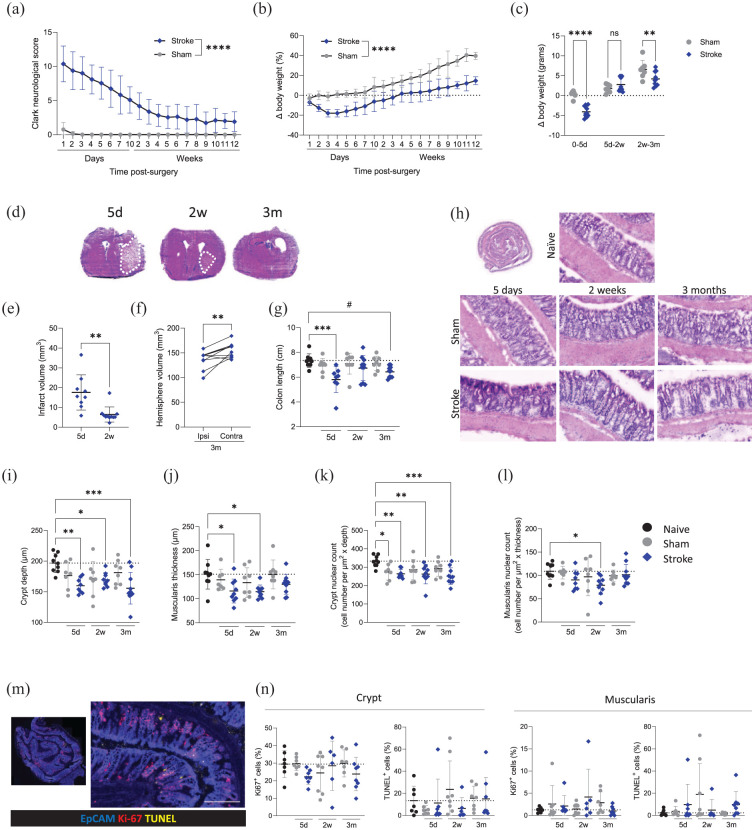
Changes to colon morphology during acute and chronic stroke recovery. Sham control (grey circles) and MCAO stroke (blue diamonds) animals were recovered for up to 3 months following surgery. (a) Clark focal neurological score and (b) body weight change measured over recovery. (c) Body weight change (g) during phases of recovery: 0–5 days, 5 days–2 weeks, 2 weeks–3 months. (d) Representative images of H&E stained brain sections at 5 days, 2 weeks and 3 months after MCAO surgery. (e) Quantification of stroke lesion volume at 5 days and 2 weeks after MCAO. (f) Quantification of ipsi- and contra-lateral hemisphere volumes at 3 months after MCAO. (g) Colon length measured upon cull (^#^*p* = 0.052). (h) Representative images of H&E stained colons. (i) Measurement of crypt depth and (j) muscularis thickness. (k) Crypt nuclear count normalised to crypt depth and (l) muscularis nuclear count normalised to thickness in H&E stained colons. (m) Representative images of immunostaining for epithelial cells (EpCAM; blue), proliferation (Ki-67; red) and cell death (TUNEL; yellow; arrows) scale bar 200 μm. (n) Measurement of the percentage of Ki-67^+^ or TUNEL positive cells in the crypt and the muscularis. Analysed by mixed-effects model with the Geisser–Greenhouse correction (a, b), unpaired *t*-tests (c, e, f) or one-way ANOVA with Dunnett’s multiple comparisons test with comparison to naive (g, i–n), *N* = 5–10/group. Data shown as mean ± SD with *p* values indicated by asterisks as follows: ****p < 0.0001, ***p < 0.001, **p < 0.01, *p < 0.05. Dotted line demarks the mean of the naive group. Data (a–n) generated from Experiment 1 (Figure S1). H&E: haematoxylin and eosin.

To investigate the intestinal microenvironment over the course of stroke recovery, animals underwent experimental stroke or sham surgery and were recovered to 5 days, 2 weeks or 3 months. Substantial neurological deficits were observed acutely after stroke with gradual recovery thereafter (Figure 1(a)). By 3 months, only mild neurological symptoms persisted, including one-sided torso flexion and assymetrical whisker reflexes. Stroke animals experienced up to 20% loss in body weight acutely and continued to lag behind sham controls (Figure 1(b) and (c)). Unexpectedly, during the mid-recovery phase (5 days–2 weeks), average weight gain was similar between sham and stroke animals. However, during the chronic phase of recovery (2 weeks–3 months), stroke animals again exhibited reduced weight gain compared to sham controls (Figure 1(c)). Resolution of the stroke lesion was examined across recovery (Figure 1(d)). Lesion volume reduced between the acute (5 days) and the sub-acute (2 weeks) time points (Figure 1(e)). At 3 months post-stroke, a lesion can no longer be detected, instead there is atrophy of tissue in the ipsilateral hemisphere resulting in increased ventricle volume and reduced hemisphere volume in comparison to the contralateral hemisphere (Figure 1(d) and (f)).

Colon length is used often used as a crude but reliable proxy measure of colonic inflammation.^33,34^ Of note, stroke led to a significant reduction in colon length (~25% shorter than those of controls). This colon shortening was also evident at 3 months post-stroke (Figure 1(g)), indicative of persistent changes to the colonic environment.

To assess macroscopic structure, H&E staining was performed on colon roll sections (Figure 1(h)). Crypt blunting (reduced crypt depth) was observed at all timepoints post-stroke (Figure 1(i)). At 5 days post-stroke, shorter colon lengths and crypt depths were associated with larger ischaemic lesions, indicating stroke severity may determine the extent of pathological change in the intestine (Figure S2(a)). However at 3 months, colon length and crypt depths showed no relationship with initial stroke severity, as measured by clark focal neurological score 2 days post-stroke (Figure S2(b)). Additionally, thinning of the muscularis in the colon was noted at 5 days and 2 weeks post-stroke, although muscularis thickness had returned to baseline levels by 3 months (Figure 1(j)). Cell numbers were reduced in the crypt at all timepoints (Figure 1(k)), however reduced cellularity was also observed at 5 days post-sham surgery suggesting the impact of recovering from surgery may play a role acutely. Reduced cellularity was observed in the muscularis only at 2 weeks post-stroke (Figure 1(l)). Despite structural changes to the colon, no clinical or histological signs of colitis were detected.

To determine if the macroscopic changes were associated with altered cell turnover, we performed immunofluorescent staining of apoptosis (TUNEL) and proliferation (marked by Ki-67 expression) within the colonic crypts and muscularis (Figure 1(m)). There was a trend of reduced Ki67^+^ cells within the crypt at 5 days post-stroke compared with naive and sham groups, but otherwise there was no evidence of altered cell proliferation or apoptosis (Figure 1(n)). As cell density within these structures is unchanged (Figure S2(c) and (d)), atrophy of the crypt and muscularis may drive the observed reduction in total cell number.

Together these findings suggest persistent alterations to intestinal morphology during chronic stroke recovery, warranting further investigation into the intestinal environment and functional capacities throughout stroke recovery.

### Stroke-induced functional impairments in the gut recover at chronic timepoints

**Figure 2.**
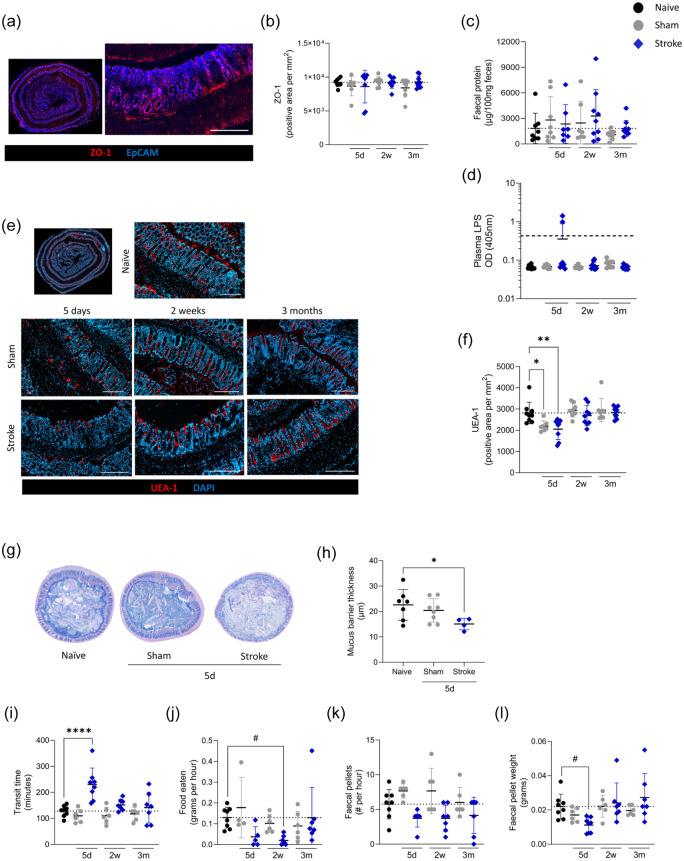
Selected intestinal functional impairments in acute stroke recovery at chronic timepoints. Colons and plasma were collected from MCAO stroke (blue diamonds) and sham (grey circles) and naive (black circles) controls mice at 5 days, 2 weeks and 3 months of recovery. (a) Colons were immunostained for tight junctions (ZO-1; red) and epithelial cells (EpCAM; blue); scalebar 200 μm. (b) Quantification of ZO-1^+^ tight junctions in colonic crypts. (c) Faecal protein concentration was measured in faecal supernatant. (d) LPS concentration quantified in plasma. (e) Representative images of colons immunostained for UEA-1 (red) and DAPI (blue) and associated (f) quantification of UEA-1. (g) Alcian blue and haematoxylin staining of colon cross-section with faecal material intact and (h) associated quantification of mucus barrier thickness. (i) Time (min) reported for orally gavaged FITC–dextran to appear in faecal pellet. (j) Amount of food (g/h) eaten (^#^*p* = 0.076) and (k) faecal pellets produced over the course of an hour. (l) Average weight of individual faecal pellet (^#^*p* = 0.060). Scalebar 200 μm (a, e) Data presented as mean ± SD with *p* values indicated by asterisks as follows: ****p < 0.0001, **p < 0.01, *p < 0.05. Dotted line is the mean of the naive group (b, c, f, i–l) or the limit of detection (d), *N* = 7–9/group (b–d, f), *n* = 4–8 (h), *n* = 6–8 (i–l). Analysed by one-way ANOVA with comparison to naive (b–d, f, h, j–l) or Kruskal–Wallis test (i). Data (a–f) generated from Experiment 1, (g, h) Experiment 4 and (i–l) Experiment 3 (Figure S1).

Next, selected intestinal functions were assessed, including barrier integrity, mucus production and transit time. To investigate gut barrier integrity we examined expression of the tight junction protein ZO-1 by colonic epithelial cells, which is known to display reduced expression in the colon in the first 24 h after experimental stroke.^35^ In agreement with studies showing resolution of barrier integrity 24 h post-experimental stroke.^11^ no changes in ZO-1 expression were detected at any of the recovery time points assessed (Figure 2(a) and (b)). There was a non-significant trend for higher infarct volumes associated with lower levels of ZO-1 immunostaining at 5 days post stroke (Figure S3(a)), however, this relationship was not apparent at 2 weeks (Figure S3(b)). Additionally, total protein in faecal supernatant was unchanged (Figure 2(c)), suggesting the outward intestinal barrier is intact at these recovery time points. Inward barrier integrity was examined by measuring bacterial LPS in plasma; only two animals at 5 days after stroke had detectable levels of circulating LPS, suggesting that the inward gut barrier is also largely intact at the time points assessed (Figure 2(d)). Next, to examine the presence of goblet cells, we performed labelling of UEA-1, a lectin that binds to fucosyl glycoconjugates on goblet cells. UEA-1 staining was reduced in both sham- and stroke-operated animals at the 5-day recovery time point. Levels returned to baseline by 2 weeks and 3 months post-stroke (Figure 2(e) and (f)). That these effects were observed in both sham-operated and stroke groups suggests that the number of goblet cells and/or mucin production may be an effect of surgery. The mucus barrier at 5 days post-stroke was further assessed in transverse colon sections, with intact faecal material (Figure 2(g)). In support of UEA-1 quantification, mucus barrier thickness was reduced in in comparison to naive controls, with sham animals demonstrating an intermediate phenotype (Figure 2(h)).

Gut transit time was assessed by measuring the time taken for gavaged FITC–dextran to appear in faeces, and was significantly delayed at 5 days post-stroke but normalised at later recovery time points (Figure 2(i)). Food intake was measured throughout the experiment and stroke animals showed a tendancy to consume less food at 5 days of recovery, which may contribute to the acute delayed transit time and reduced weight of stroked animals (Figure 2(j)). However, at 2 weeks post-stroke, food intake remained reduced but transit time and loss of body weight were similar to controls, suggesting additional factors may contribute to these phenotypes (Figure 1(c)). In agreement with data on food intake, the number of faecal pellets produced per hour (Figure 2(k)) and the faecal weight of pellets produced (Figure 2(l)) were also reduced early after stroke.

Together these data demonstrate that certain functional alterations occur acutely after stroke and do not persist into chronic recovery. Additionally, changes to mucus barrier functions, food intake and gut transit time may contribute to intestinal dysfunction early after stroke but are unlikely to contribute to any persistant alterations to intestinal homeostasis.

### Stroke induces dynamic changes to the microbiome which persist into chronic recovery

Shifts in the composition of the intestinal microbiota is well reported during acute stroke recovery in both animal models and human studies.^36,37^ To profile the community composition induced by our model of stroke, we performed 16S rRNA sequencing of faecal material from naive and stroke animals. In these experiments, co-housing of stroke animals with naive or sham-operated controls resulted in an overlapping community composition between treatment groups (Figure S4(a) and (c)). However, when animals were separately housed, the microbiome of naive and shams were distinct from that of stroke animals (Figure S4(b) and (d)). For this reason, stroke samples were analysed in comparison to baseline samples, comprised of samples taken prior to surgery and from naive animals that were maintained separately, to minimise bias introduced by cage and co-housing. Additionally, these data are comprised of two independent experiments where colon content samples were harvested at individual timepoints (Experiment 1; Figure S1) or stool were collected across the recovery timecourse (Experiment 2; Figure S1). Although some variation due to sample type was apparent, predominantly in the naive animals (Figure S4(e)), analysis of bacterial phyla in both colon content and stool demonstrated stroke-induced alterations to phyla relative abundance was similar (Figure S4(f) and (g)), thus data from both experiments were compiled for increased analytical power.

**Figure 3.**
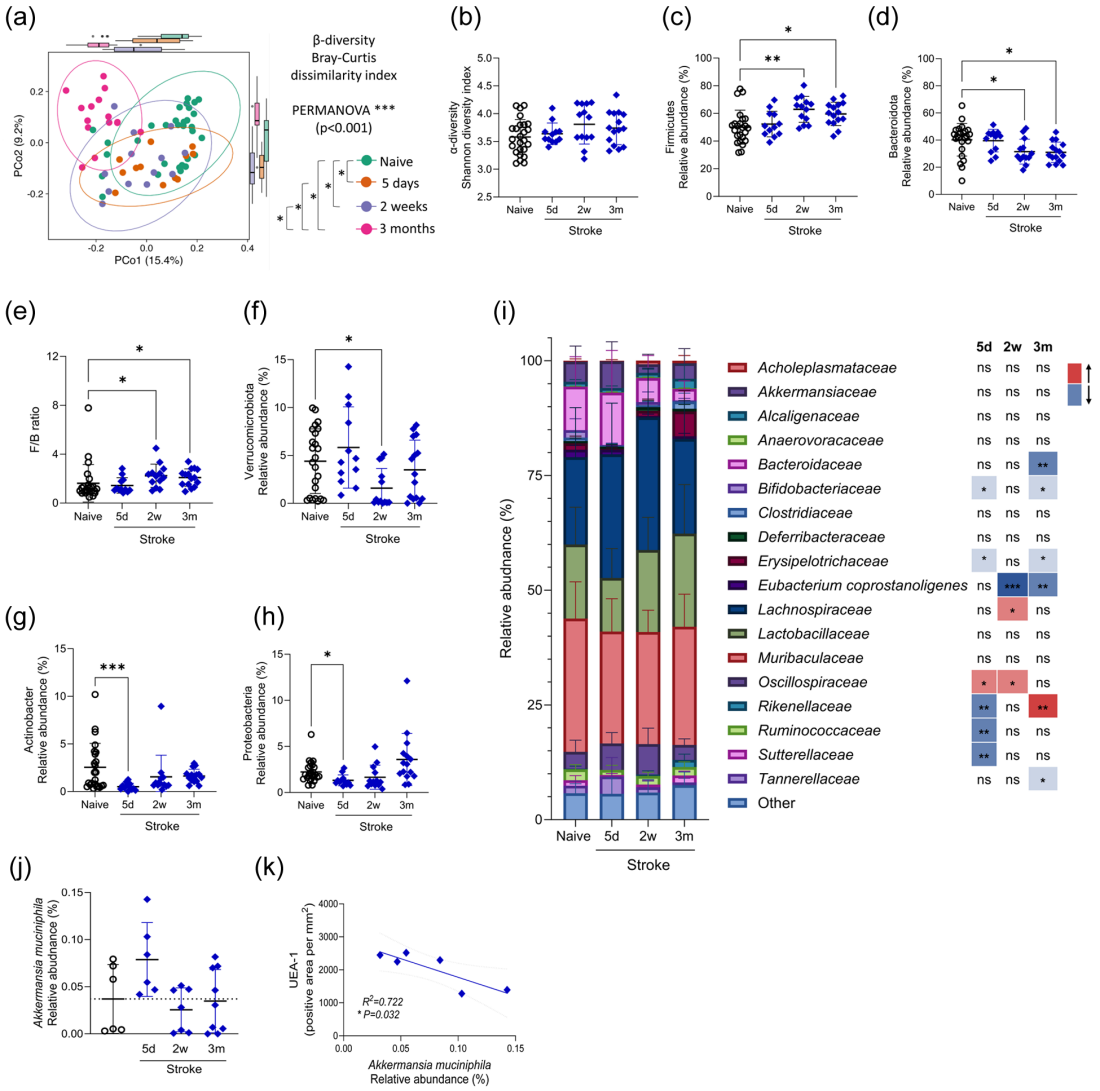
Stroke induces dynamic changes to the microbiome which persist into chronic recovery. Sham (grey circles) and naive (black circles) control, and MCAO stroke (blue diamonds) animals were recovered for up to 3 months following surgery and 16S rRNA sequencing was performed on faecal material. Naive samples were obtained pre-surgery or from naive mice were were not co-housed with surgical mice. (a) Bray–Curtis dissimilarity index, analysis during stroke recovery and (b) Shannon diversity index. (c–h) Relative abundance of various phyla, including Firmicutes, Bacteroidota, Verrucomicrobiota, Actinobacter and Proteobacteria and the ratio of Firmicutes to Bacteroidota. (i) Relative abundance of bacterial families with an abundance of >1% of total population. Bacterial families comprising <1% were included in the group defined as other. (j) Relative abundance (%) of *Akkermansia muciniphila* during stroke recovery. (k) Correlation between area of UEA-1 immunostaining and *Akkermansia mucinphila* relative abundance at 5 days post-stroke. Data presented as mean ± SD (a, c–j) or 95% confidence interval (b) with *p* values indicated by asterisks as follows: ***p < 0.0001, **p < 0.01, *p < 0.05. Data are pooled from two independent experiments, *n* = 12–25. Data analysed by one-way ANOVA, or Kruskal–Wallis test if SD’s significantly different by the Brown–Forsythe test, with comparison to naive (b–h, j), PERMANOVA, with and without multiple comparisons test (a), mixed-effects analysis with Dunnett’s multiple comparisons test with comparison to baseline (i) or linear regression with best fit line plotted (k), *N* = 12–25 (a–h) or *n* = 6–8 (j, k) per group. Data generated from combined analysis of Experiments 1 and 2 (a–h) or from Experiment 1 (j, k; Figure S1).

The microbial community composition was significantly altered between naive and stroke sample groups at all timepoints. Interestingly, community composition was different between early (5 days and 2 weeks) and late (3 months) recovery timepoints (Figure 3(a)). These results suggest that stroke affects the intestinal microbiota throughout recovery in a dynamic manner, with distinct compositions observed during acute versus chronic recovery.

The diversity within the microbial community of individual samples was measured with the Shannon diversity index and shown to be unchanged throughout stroke recovery (Figure 3(b)), demonstrating the observed dissimilarity between sample groups is not due to altered total species evenness and richness. Therefore, we began investigating the relative abundance of various bacterial species.

Firmicutes and Bacteroidetes are the two most dominant phyla of the mouse gut microbiota^38^ and their ratio has been linked to various conditions in humans including obesity,^39^ cardiovascular disease^40^ and sepsis.^41^ Significant increases in the abundance of Firmicutes (Figure 3(c)) and reductions in Bacteroidota (Figure 3(d)) were detected at 2 weeks and 3 months after stroke resulting in altered Firmicutes/Bacteroidota ratios at these time points in comparison to naive controls (Figure 3(e)). Further changes detected at the phylum level included reduced abundance of Verrucomicrobiota at 2 weeks (Figure 3(f)), and reduced Actinobacter (Figure 3(g)) and Proteobacteria (Figure 3(h)) at 5 days post-stroke, in comparison to naive controls.

Investigation of gut microbiome at the family level further demonstrated different temporal trajectories. The abundance of some families showed changes only at 5 days (*Ruminococcacea, Sutterellaceae)* or 3 months (*Bacteroidaceae, Tanerellaceae*) post-stroke. Other families showed opposing changes in abundance at 5-day and 3-month recovery timepoints (*Rikenellaceae*) whereas others showed changes at 5-day time points which returned to baseline at 2 weeks and reoccurred at 3 months (*Bifidobacteriaceae, Erysipelotrichaseae*; Figure 3(i)). We explored the impact of the initial stroke severity on the extent of microbiome change and found no relationship with the abundance of Actinobacter at 5 days (Figure S4(h)) or Firmicutes at 3 months post-stroke (Figure S4(i)).

Lastly, we performed exploratory analysis to determine any bacterial species alterations may relate to observed structural or functional alterations. Of note, *Akkermansia muciniphila*, known to degrade mucins, showed increased relative abundance at 5 days post-stroke (Figure 3(j)). Relative abundance of *A. muciniphila* inversely correlated with colonic UEA-1 immunostaining at 5 days post-stroke (Figure 3(k)), but not at 2 weeks or 3 months (Figure S4(j) and (k)).

In summary, the intestinal microbiome displayed dynamic changes throughout stroke recovery. Distinct microbial alterations during acute and chronic timepoints suggest that different factors may be driving microbiota changes across recovery. Reduced eating (Figure 2(j)), impaired transit (Figure 2(i)) and reduced mucus barrier (Figure 2(h)) may contribute to population changes measured during acute stroke recovery but these are unlikely to be associated with chronic microbial alterations.

### Disrupted intestinal immune homeostasis occurs throughout stroke recovery

Intestinal homeostasis is crucial for normal physiological and barrier functioning and is tightly regulated by a number of host response mechanisms. Shifts towards inflammation or immunosuppression can disrupt intestinal morphology, functionality and bacterial community composition. Following stroke, immune homeostasis is disrupted in various peripheral tissues, with a combination of hyporesponsiveness and inflammation observed.^42^ Given this, we next measured soluble immune factors in the intestinal lumen over recovery.

**Figure 4.**
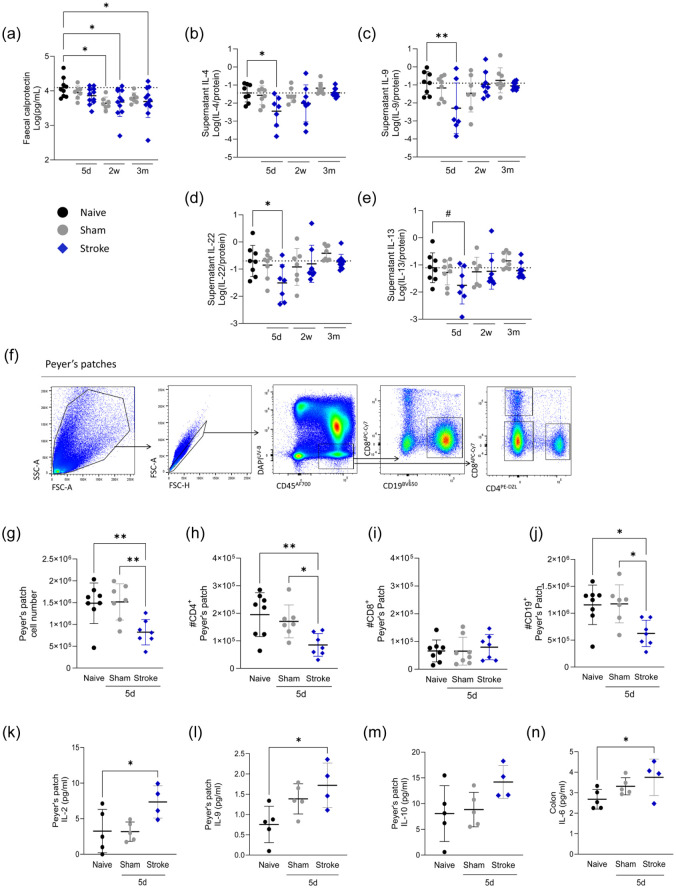
Disrupted intestinal immune homeostasis throughout stroke recovery. (a) Calprotectin was measured by ELISA in faecal supernatant from sham (grey circles) and naive (black circles) control and MCAO stroke (blue diamonds) animals. (b–e) Concentrations of faecal supernatant cytokines (pg/ml) normalised to protein (μg/ml). (f) Representative contour plot for identification of lymphocytes in PP. (g) Total live cell counts of PP and (h) CD4^+^ T cell, (i) CD8^+^ T cell and (j) CD19^+^ B cell numbers in PP from sham and naive control and MCAO stroke animals 5 days post-stroke. Concentration of PP (k–m) and colon (n) cytokines (pg/ml) significantly increased at 5 days post-stroke. (a–e, g–n) Data shown as mean ± SD. If data was not normally distributed by Shapiro–Wilk it was log-transformed. Data analysed by one-way ANOVA with Dunnett’s multiple comparisons test, comparison to naive, *N* = 7–10/group (a–e), *n* = 7–8/group (g–j), *n* = 4–5/group (k–n). Data shown as mean ± SD with *p* values indicated by asterisks as follows: **p < 0.01, *p < 0.05, # *p* = 0.079. Dotted line is the mean of the naive group. Data (a–e) generated from Experiment 1, (f–j) generated from Experiment 4 and (k–n) generated from Experiment 5 (Figure S1). PP: Peyer’s patch.

The level of faecal calprotectin, a proinflammatory factor produced by neutrophils and monocytes, was reduced at 2 weeks and 3 months post-stroke (Figure 4(a)). Expanding on this, we measured colonic luminal cytokines and found reductions in concentrations of IL-4, IL-9 and IL-22 at 5 days after stroke, with a similar trend observed for IL-13 (Figure 4(b)–(e)). In exploratory analysis, lower concentrations of IL-4, IL-9 and IL-13 were present in animals with larger infarcts, but this relationship was not seen with IL-22 (Figure S5(a)). In contrast, concentration of IFNγ, TNF, IL-2, IL-17F, IL-5, IL-6, IL-17A and IL-10 remained relatively unchanged throughout recovery (Figure S5(b)). No increase in luminal cytokine concentrations were detected in the faecal supernatant at any time point.

Acute loss of intestinal immune cells, specifically in the Peyer’s patches (PP), has been described after stroke.^18,19,21,43^ In agreement, we saw reduced PP cellularity, driven by a loss of CD4 T cells and B cells at 5 days post-stroke (Figure 4(f)–(j)) and the proportion of intraepithelial and lamina propria lymphocytes remained unchanged (Figure S5(c)–(g)). It has been reported that T cells can migrate from the intestine to the brain in the first week after stroke.^19^ Although we cannot confirm the origin, we observed an accumulation of T cells in the brain, specifically within the ipsilateral hemisphere (Figure S5(h) and (i)), that coincided with reduced T cells in the PP (Figure 4(f)–(h)).

To determine if reduced luminal cytokines may be driven by a loss of lymphocytes in the PP, we measured cytokine concentration in PP and colon tissue homogenate 5 days after stroke. We found that concentration of IL-2 and IL-9 were significantly increased in PP after stroke, with IL-10 concentrations showing a similar trend (Figure 4(k)–(m)). IL-6 was specifically increased in the colon tissue (Figure 4(n)). All other cytokines measured in PP (Figure S6(a)) and colon (Figure S6(b)) remained unchanged from baseline.

Together, these data demonstrate that experimental stroke exerts both temporal and spatial alterations to the intestinal cytokine mileu, with anatomical microenvironments of the intestine displaying distinct immunological profiles.

### Altered antibody coating of intestinal bacteria in chronic stroke recovery

**Figure 5.**
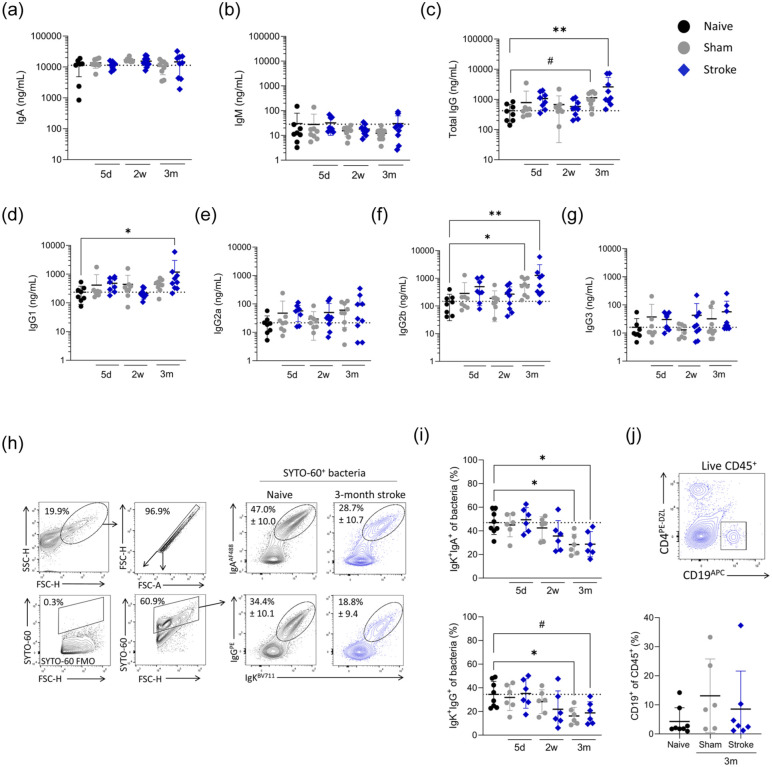
Altered antibody coating of intestinal bacteria in chronic stroke recovery. Faecal samples were processed for faecal supernatant and a bacterial fraction, from sham (grey circles) and naive (black circles) control and MCAO stroke (blue diamonds) animals. (a–g) Concentration of antibody isotypes (ng/ml) in faecal supernatant. (h) Representative contour plots of gating strategy for luminal bacteria and IgA^+^IgK^+^ and IgG^+^IgK^+^ bacteria from representative samples and (i) associated quantification. (j) Flow cytometry plots from lamina propria fractions of colon and associated quantification of B cells (%CD19^+^ of CD45^+^). Data analysed by one-way ANOVA with Dunnett’s multiple comparisons test, comparison to naive, *N* = 7–10/group (a–g), *n* = 6–8/group (i, j). Data shown as mean ± SD with *p* values indicated by asterisks as follows: **p < 0.01, *p < 0.05, # *p* = 0.059. Dotted line is the mean of the naive group. Data (a–i) generated from Experiment 1, (j) generated from Experiment 4 (Figure S1).

Intestinal antibodies, in particular immunoglobulin A (IgA), are key regulators of the microbial community composition.^44–46^ Stroke can cause reductions in circulating antibodies in both patients^47^ and experimental animal models.^48^ We measured antibody concentrations in faecal supernatant to determine if luminal antibody concentrations were affected by stroke. The concentration of luminal IgA, the most abundant antibody isotype in the gut, remained unchanged following stroke (Figure 5(a)). Similarly, IgM levels showed no significant changes (Figure 5(b)). In contrast, we observed a notable increase in IgG concentrations in the faecal supernatant 3 months post-stroke (Figure 5(c)), driven primarily by increases in IgG1, IgG2b and IgG3 isotypes (Figure 5(d)–(g)).

Alterations in the antibody coating of intestinal bacteria have been linked to acute post-stroke dysbiosis.^18,43^ To understand if increased IgG in the intestinal lumen observed at 3 months post-stroke altered the antibody coating of intestinal bacteria, flow cytometry was performed on bacteria isolated from faecal pellets (Figure 5(h)). Surprisingly, at this timepoint, where faecal IgG was elevated, bacteria showed less surface coating of both IgA and IgG (Figure 5(i)). Reduced antibody coating was also seen on bacteria isolated from sham-operated animals, and as these animals were co-housed with stroked cagemates and exhibited the same gut microbiome composition (Figure S4(c)), this suggests that the type of bacteria present may drive reduced bacterial coating, rather than the availability of antibody. Indeed, a trend towards increased luminal IgG concentration was also seen in sham-operated animals at 3 months (Figure 5(c)). Furthermore, no increase in lamina propria B cells, the major source of antibody secreted into the intestinal lumen, was observed (Figure S5(f) and (g)).

These results, in combination with altered cytokine profiles, highlight that the immunological environment in the intestine is altered during both acute and chronic recovery. The coexistence of increased unbound IgG and increased bacteria-bound IgG suggests potential interplay between antibody binding and availbility and bacterial community composition during chronic stroke recovery.

## Discussion

Post-stroke intestinal dysfunction has been described during acute recovery and a relationship between the intestinal environment and the recovering stroke brain is clear. Here, we build on these findings with a longitudinal analysis that demonstrates changes to intestinal homeostasis take place over the course of recovery. To our knowledge, this is the first description of intestinal alterations and microbial community chages during chronic stroke recovery. Importantly, these changes are dynamic, with early resolution of functional deficits, while both bacterial community composition and immunological environment displaying distinct profiles during acute and chronic phases of recovery. Although causation cannot be inferred from the current study, we provide insight into possible interplay between immunological factors and microbial community composition in the intestine throughout stroke recovery.

Histological analyses focussed on the colon as this carries the highest bacterial burden across the intestine^49^ and a shortened colon can be used as a proxy measure of colonic inflammation.^33,34^ We observed blunted crypt depth with reduced crypt cellularity throughout the recovery timecourse. Epithelial cell death, induced by activation of sympathetic nerves, has been reported to occur throughout the intestine in hyperacute recovery from experimental stroke^11^ and combined with the trend for reduced proliferation we observed during acute recovery, may contribute to these changes. However, factors that contribute to prolonged changes to colon morphology are yet to be elucidated. Changes to the functional capacity of the gut have been widely described during hyperacute recovery from stroke. We show that gut barrier function is intact at day 5 and remains intact therein, in agreement with published data showing integrity restoration as early as 24 h post-stroke.^9,11,43^ We found gut transit time remained impaired at 5 days but was restored in chronic recovery. Recent studies have shown that impaired peristalsis is driven by altered neural output in the gut after stroke^10^ and reduced mucin production may also play a role in delayed transit times.^50,51^ In our studies, reduced food intake was observed acutely, and may further contribute to a prolonged transit time. As functional impairments and reduced food intake are confined to acute recovery, it is plausible that these are important factors in the distinct microbiota alterations observed at this time point, although further studies would be required to confirm this.

Firmicutes and Bacteroidetes are the two most dominant phyla in both humans^52^ and mice^38^ and the ratio of their abundance (F:B ratio) has been assessed in association with several pathological conditions including obesity,^39^ type 2 diabetes,^53^ and cardiovascular disease.^54^ Alterd F:B ratio occurs acutely after experimental stroke^55^ which, we have shown increase in magnitude of change as recovery progresses. Firmicutes is reported to demonstrate reduced antibody surface coating than other enteric flora phyla^56,57^ and the expansion of intestinal firmicutes over time may drive the overall reduction in bacterial antibody coating found at chronic time points, independently of antibody availability.

We chose to co-house the stroked animals with controls, in line with the IMPROVE Guidelines, to enhance their welfare throughout the chronic recovery experiments.^26^ Mice are corporophagic and seeder mice can be used experimentally to modify the microbiome of their cagemates.^58^ We observed that the microbiome of sham animals took on the profile of co-housed stroke animals, meaning that sham-induced alterations in intestinal homeostasis from naive controls may not only be attributable to the surgical procedure. This experimental set up can aid hypothesis generation about cause and consequence when comparing dysbiosis alongside changes to the intestinal microenvironment.. Together, these associations should allow future hypothesis-driven research to understand the precise mechanisms of gastrointestinal disturbances, with a particular focus on antibody coating of bacteria, during chronic stroke recovery and pave the way for potential therapeutic intervention.

Sharing of the microbiome with co-housed controls raises debate about experimental design and co-housing in stroke experiments. Many selective pressures can influence microbiome composition, including diet, mucus content, luminal IgA, antimicrobial peptides and microRNAs.^59^ Continuous exposure of cagemates microbiome through coprophagic behaviour may provide an ongoing source of more dominant microbial families, or drive local or systemic immune changes which maintain these microbiome changes. Co-housing with sick animals can induce stress in healthy cage mates via activation of the HPA axis and sympathetic nervous system,^60,61^ stress pathways known to drive systemic immune changes after stroke. We explored the impact of initial stroke severity on microbial changes but did not see an association. This may be due to a shared microbiome between co-housed stroked animals, masking any initial impact of stroke severity in individual animals.

Experimental factors that affect microbiome composition include stress from regular handling of animals. In these experiments, sham and stroke animals were neurologically scored, whereas naive animals were not. However, when sham animals are not co-housed with stroked cagemates, they retain a microbiome profile similar to that of naive animals, suggesting handling stress has minimal impact on microbial diversity in these studies. The relative abundance of bacterial phyla was similar between stool samples and colon content, which contrasts with human studies demonstarating these sample types can give distinct microbial profiles.^62–64^ Our data suggests, the stroke microbial phenotype was shared with control cage mates, perhaps in the context of disease, differences across the intestine and between samples are reduced. However, PCoA analysis showed samples clustered by experimental time point, but some separation by sample type was also apparent, particularly in the naive group, showing some influence of sample source on microbiota profiles.

Immunological changes, notably cytokine profiles, were altered both temporally and spatially in the intestine after stroke. Although PP cellularity was reduced acutely after stroke, we saw an increase in PP IL-2 and IL-9, cytokines important for lymphocyte proliferation and survival.^65,66^ In the colon, only IL-6 was elevated acutely after stroke, which is a known driver of structural alterations and pathology in inflammatory bowel disease.^67^ Differences in innervation across the gut^68^ may explain microenvironment specific changes to the cytokine mileu across the intestine after stroke. In contrast, IL-4, IL-9 and IL-22 were reduced in the intestinal lumen. Intestinal microbiota can both induce cytokine responses in immune cells, but also inhibit cytokine synthesis or release, and may be a more predominant driver of changes to immunological profile within the intestinal lumen. Together these data highlight the complexity of the intestinal environment after stroke wherein microanatomical location can determine the signals received after stroke and the resultant phenotype.

A caveat of this study is that data were aquired from young, healthy, male mice and factors such as age, sex and co-morbidities are not accounted for. Indeed, studies using aged mice have shown that intestinal inflammation and barrier dysfunction are exacerbated with age.^35^ In addition, there are discrepancies in the literature on whether goblet cells and mucin production increase or decrease after stroke.^11^ Time of sampling post-stroke may in part account for this, however sex differences may play an important role as increased mucus production out to 7 days after stroke was found to be specific to females, with males instead increasing their production of anti-microbial peptides.^69^ Future studies looking at chronic intestinal disturbance in aged, mixed sex and co-morbid mice are required to expand on the initial insights we provide. However, as these factors can independently influence the intestinal environment, it is important to know the impact stroke, which itself imparts a complex mix of signals on the intestine, prior to adding complicating factors. Despite the need for further studies, our initial data suggests that stroke results in persistent changes in gut physiology along with altered antibody availability and microbial composition.

## Conclusions

This study provides foundational understanding into the evolution of gastrointestinal disturbances after stroke. The data presented here demonstrate that experimental stroke induces significant alterations in the intestinal environment, with these changes evolving dynamically across time and peristing into the chronic recovery phase. Here, we demonstrate that the intestinal microbiota and immunoloigical profile is altered during chronic recovery, though whether these confer vulnerability or protection to additional insults, such as enteric infection, or modulate subsequent neurological insults, such as recurrent stroke, is unclear. Further studies will be required to determine whether persistent changes to these microbial families have a direct role in conferring poorer stroke outcomes throughout recovery.

## Supplemental Material

sj-pptx-1-jcb-10.1177_0271678X251405669 – Supplemental material for Dynamic changes to the intestinal environment occur throughout recovery from experimental ischaemic stroke Supplemental material, sj-pptx-1-jcb-10.1177_0271678X251405669 for Dynamic changes to the intestinal environment occur throughout recovery from experimental ischaemic stroke by Rachel M L Martin, Isobel C Mouat, Robert J Whelan, Lizi M Hegarty, Lizzie F R Blunt, Christopher J Anderson, David H Dockrell, Calum C Bain, Gwo-Tzer Ho and Laura McCulloch in Journal of Cerebral Blood Flow & Metabolism

sj-pptx-2-jcb-10.1177_0271678X251405669 – Supplemental material for Dynamic changes to the intestinal environment occur throughout recovery from experimental ischaemic stroke Supplemental material, sj-pptx-2-jcb-10.1177_0271678X251405669 for Dynamic changes to the intestinal environment occur throughout recovery from experimental ischaemic stroke by Rachel M L Martin, Isobel C Mouat, Robert J Whelan, Lizi M Hegarty, Lizzie F R Blunt, Christopher J Anderson, David H Dockrell, Calum C Bain, Gwo-Tzer Ho and Laura McCulloch in Journal of Cerebral Blood Flow & Metabolism
