# Supplementary material for: Dynamic changes to the intestinal environment occur throughout recovery from experimental ischaemic stroke
Source: J Cereb Blood Flow Metab. 2026 Jan 17:0271678X251405669. Online ahead of print. doi: 10.1177/0271678X251405669 (PMC12812060; doi:10.1177/0271678X251405669)
Supplement: sj-pptx-1-jcb-10.1177_0271678X251405669 – Supplemental material for Dynamic changes to the intestinal environment occur throughout recovery from experimental ischaemic stroke [file sj-pptx-1-jcb-10.1177_0271678X251405669.pptx]

## Slide 1
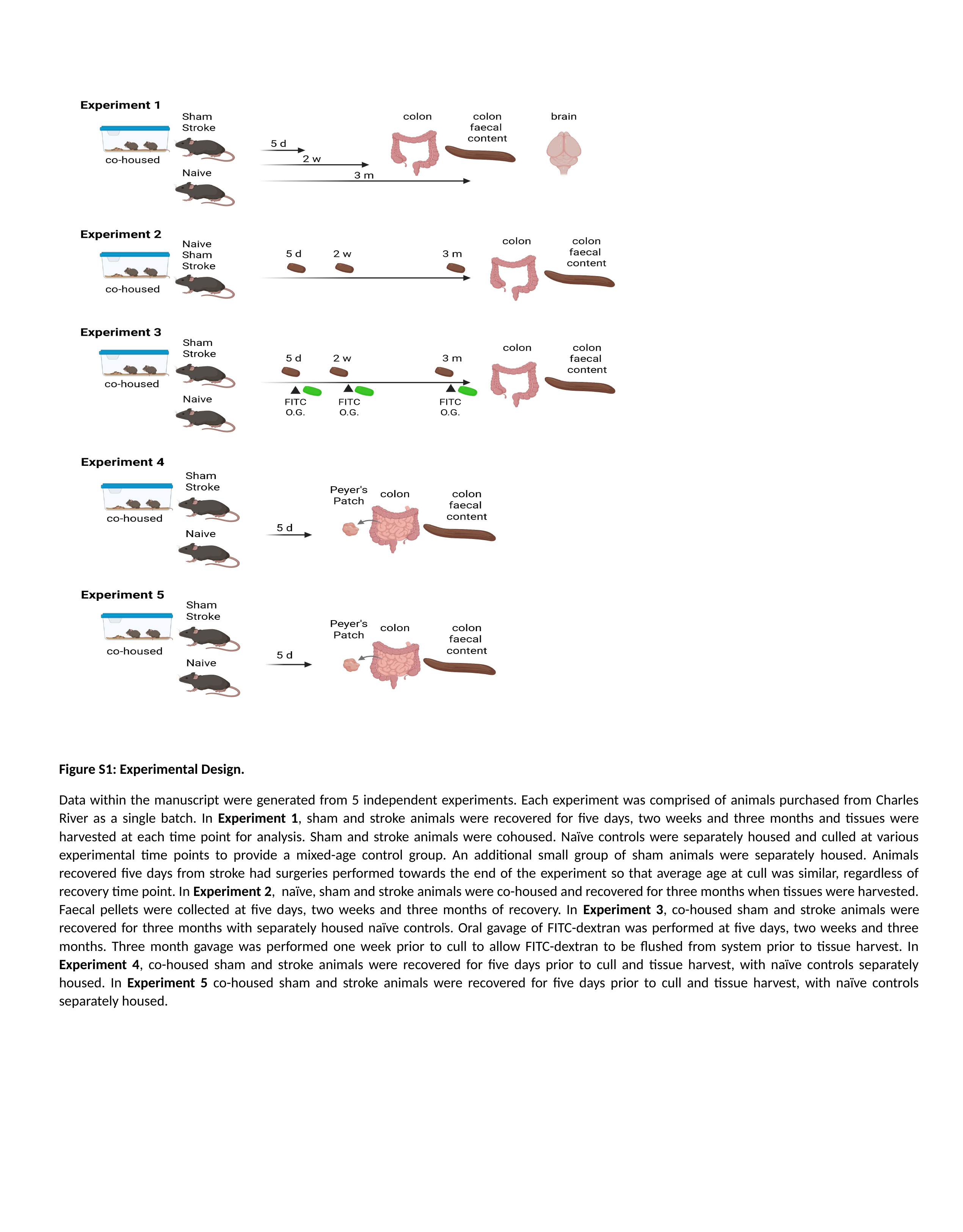

Figure S1: Experimental Design.
Data within the manuscript were generated from 5 independent experiments. Each experiment was comprised of animals purchased from Charles River as a single batch. In Experiment 1, sham and stroke animals were recovered for five days, two weeks and three months and tissues were harvested at each time point for analysis. Sham and stroke animals were cohoused. Naïve controls were separately housed and culled at various experimental time points to provide a mixed-age control group. An additional small group of sham animals were separately housed. Animals recovered five days from stroke had surgeries performed towards the end of the experiment so that average age at cull was similar, regardless of recovery time point. In Experiment 2, naïve, sham and stroke animals were co-housed and recovered for three months when tissues were harvested. Faecal pellets were collected at five days, two weeks and three months of recovery. In Experiment 3, co-housed sham and stroke animals were recovered for three months with separately housed naïve controls. Oral gavage of FITC-dextran was performed at five days, two weeks and three months. Three month gavage was performed one week prior to cull to allow FITC-dextran to be flushed from system prior to tissue harvest. In Experiment 4, co-housed sham and stroke animals were recovered for five days prior to cull and tissue harvest, with naïve controls separately housed. In Experiment 5 co-housed sham and stroke animals were recovered for five days prior to cull and tissue harvest, with naïve controls separately housed.

## Slide 2
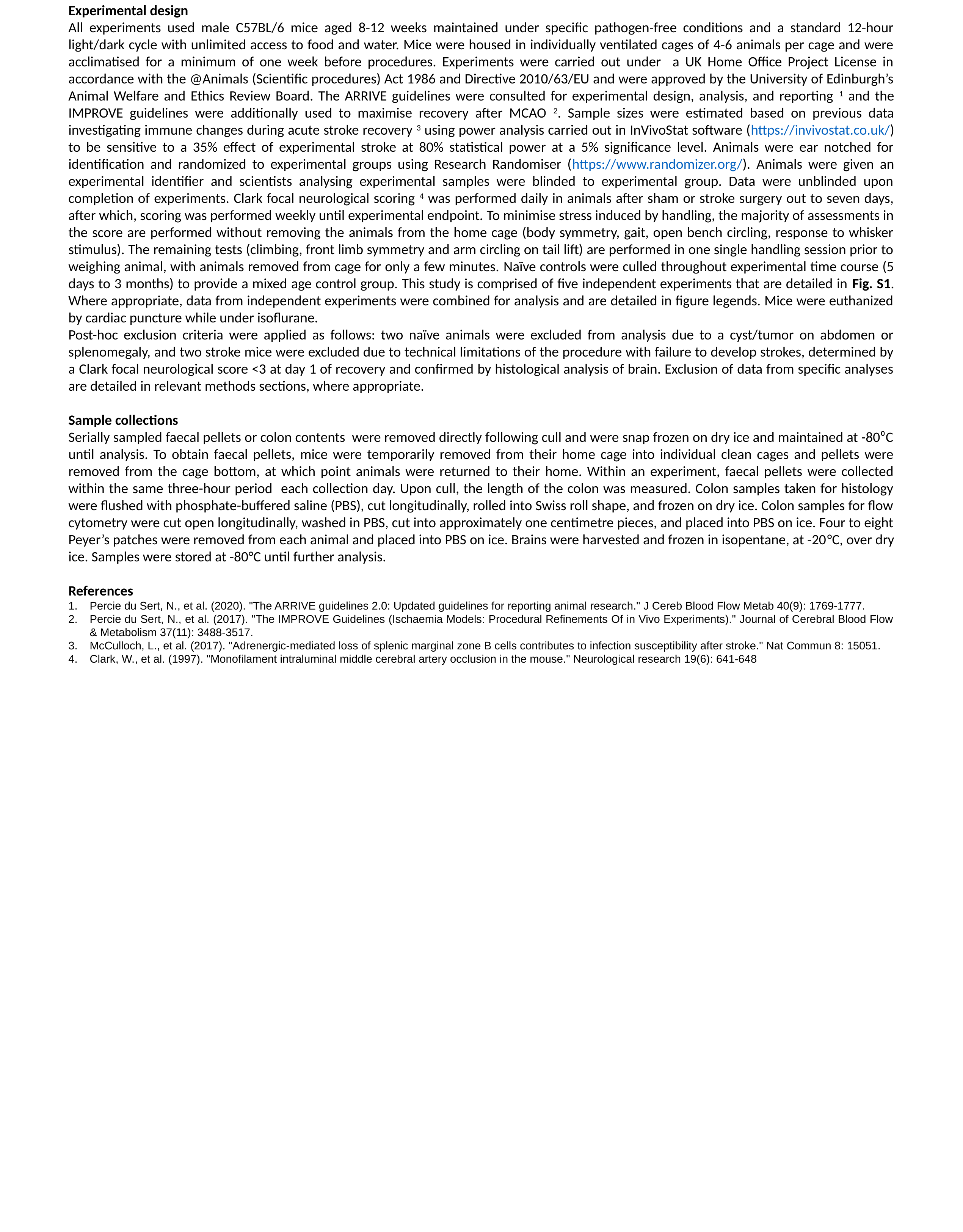

Experimental design
All experiments used male C57BL/6 mice aged 8-12 weeks maintained under specific pathogen-free conditions and a standard 12-hour light/dark cycle with unlimited access to food and water. Mice were housed in individually ventilated cages of 4-6 animals per cage and were acclimatised for a minimum of one week before procedures. Experiments were carried out under a UK Home Office Project License in accordance with the @Animals (Scientific procedures) Act 1986 and Directive 2010/63/EU and were approved by the University of Edinburgh’s Animal Welfare and Ethics Review Board. The ARRIVE guidelines were consulted for experimental design, analysis, and reporting 1 and the IMPROVE guidelines were additionally used to maximise recovery after MCAO 2. Sample sizes were estimated based on previous data investigating immune changes during acute stroke recovery 3 using power analysis carried out in InVivoStat software (https://invivostat.co.uk/) to be sensitive to a 35% effect of experimental stroke at 80% statistical power at a 5% significance level. Animals were ear notched for identification and randomized to experimental groups using Research Randomiser (https://www.randomizer.org/). Animals were given an experimental identifier and scientists analysing experimental samples were blinded to experimental group. Data were unblinded upon completion of experiments. Clark focal neurological scoring 4 was performed daily in animals after sham or stroke surgery out to seven days, after which, scoring was performed weekly until experimental endpoint. To minimise stress induced by handling, the majority of assessments in the score are performed without removing the animals from the home cage (body symmetry, gait, open bench circling, response to whisker stimulus). The remaining tests (climbing, front limb symmetry and arm circling on tail lift) are performed in one single handling session prior to weighing animal, with animals removed from cage for only a few minutes. Naïve controls were culled throughout experimental time course (5 days to 3 months) to provide a mixed age control group. This study is comprised of five independent experiments that are detailed in Fig. S1. Where appropriate, data from independent experiments were combined for analysis and are detailed in figure legends. Mice were euthanized by cardiac puncture while under isoflurane.
Post-hoc exclusion criteria were applied as follows: two naïve animals were excluded from analysis due to a cyst/tumor on abdomen or splenomegaly, and two stroke mice were excluded due to technical limitations of the procedure with failure to develop strokes, determined by a Clark focal neurological score <3 at day 1 of recovery and confirmed by histological analysis of brain. Exclusion of data from specific analyses are detailed in relevant methods sections, where appropriate.
Sample collections
Serially sampled faecal pellets or colon contents were removed directly following cull and were snap frozen on dry ice and maintained at -80⁰C until analysis. To obtain faecal pellets, mice were temporarily removed from their home cage into individual clean cages and pellets were removed from the cage bottom, at which point animals were returned to their home. Within an experiment, faecal pellets were collected within the same three-hour period each collection day. Upon cull, the length of the colon was measured. Colon samples taken for histology were flushed with phosphate-buffered saline (PBS), cut longitudinally, rolled into Swiss roll shape, and frozen on dry ice. Colon samples for flow cytometry were cut open longitudinally, washed in PBS, cut into approximately one centimetre pieces, and placed into PBS on ice. Four to eight Peyer’s patches were removed from each animal and placed into PBS on ice. Brains were harvested and frozen in isopentane, at -20°C, over dry ice. Samples were stored at -80°C until further analysis.
References
Percie du Sert, N., et al. (2020). "The ARRIVE guidelines 2.0: Updated guidelines for reporting animal research." J Cereb Blood Flow Metab 40(9): 1769-1777.
Percie du Sert, N., et al. (2017). "The IMPROVE Guidelines (Ischaemia Models: Procedural Refinements Of in Vivo Experiments)." Journal of Cerebral Blood Flow & Metabolism 37(11): 3488-3517.
McCulloch, L., et al. (2017). "Adrenergic-mediated loss of splenic marginal zone B cells contributes to infection susceptibility after stroke." Nat Commun 8: 15051.
Clark, W., et al. (1997). "Monofilament intraluminal middle cerebral artery occlusion in the mouse." Neurological research 19(6): 641-648
