# Supplementary material for: Dynamic changes to the intestinal environment occur throughout recovery from experimental ischaemic stroke
Source: J Cereb Blood Flow Metab. 2026 Jan 17:0271678X251405669. Online ahead of print. doi: 10.1177/0271678X251405669 (PMC12812060; doi:10.1177/0271678X251405669)
Supplement: sj-pptx-2-jcb-10.1177_0271678X251405669 – Supplemental material for Dynamic changes to the intestinal environment occur throughout recovery from experimental ischaemic stroke [file sj-pptx-2-jcb-10.1177_0271678X251405669.pptx]

## Slide 1
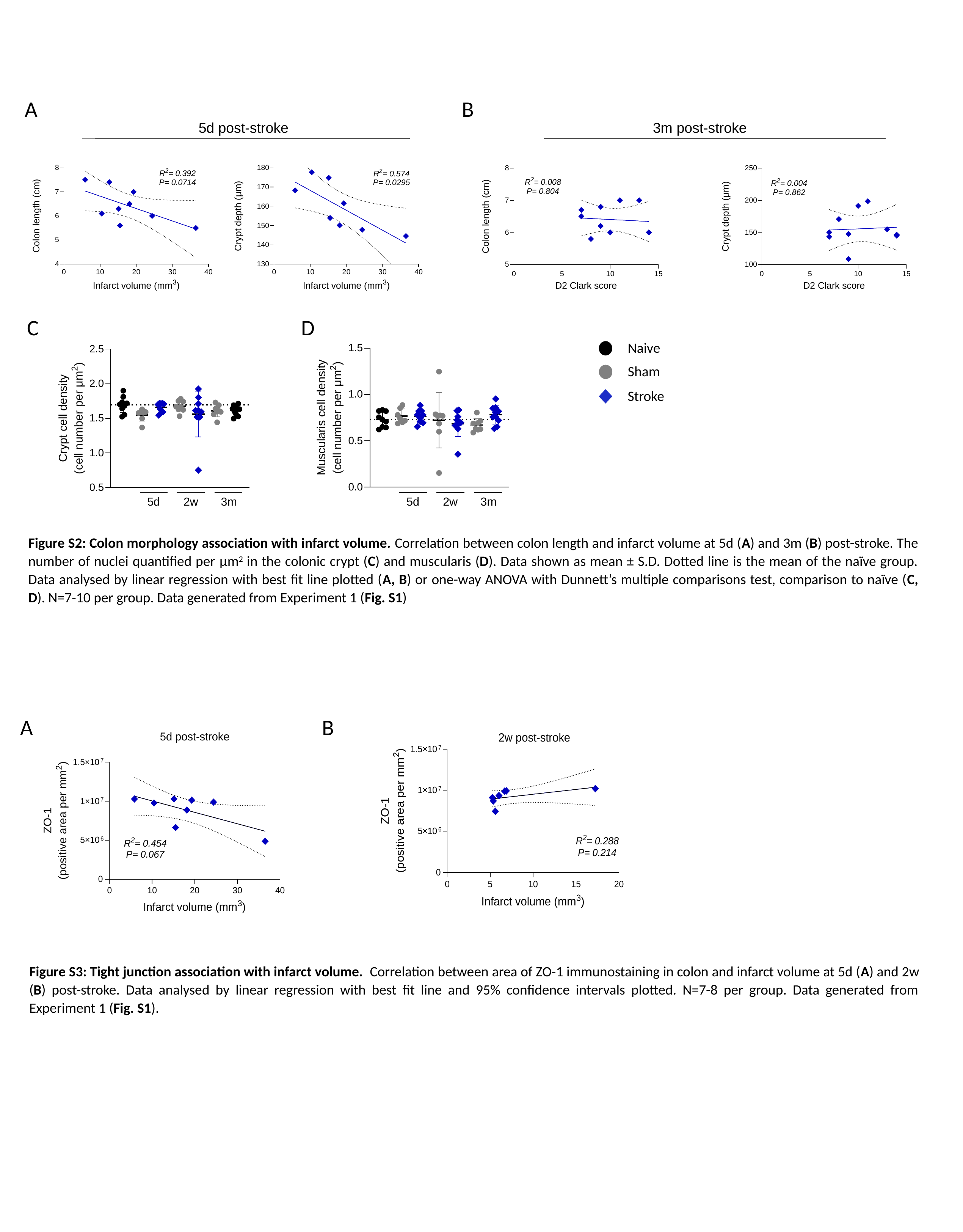

A
B
3m post-stroke
5d post-stroke
C
D
Naive
Sham
Stroke
Figure S2: Colon morphology association with infarct volume. Correlation between colon length and infarct volume at 5d (A) and 3m (B) post-stroke. The number of nuclei quantified per μm2 in the colonic crypt (C) and muscularis (D). Data shown as mean ± S.D. Dotted line is the mean of the naïve group. Data analysed by linear regression with best fit line plotted (A, B) or one-way ANOVA with Dunnett’s multiple comparisons test, comparison to naïve (C, D). N=7-10 per group. Data generated from Experiment 1 (Fig. S1)
A
B
Figure S3: Tight junction association with infarct volume. Correlation between area of ZO-1 immunostaining in colon and infarct volume at 5d (A) and 2w (B) post-stroke. Data analysed by linear regression with best fit line and 95% confidence intervals plotted. N=7-8 per group. Data generated from Experiment 1 (Fig. S1).

## Slide 2
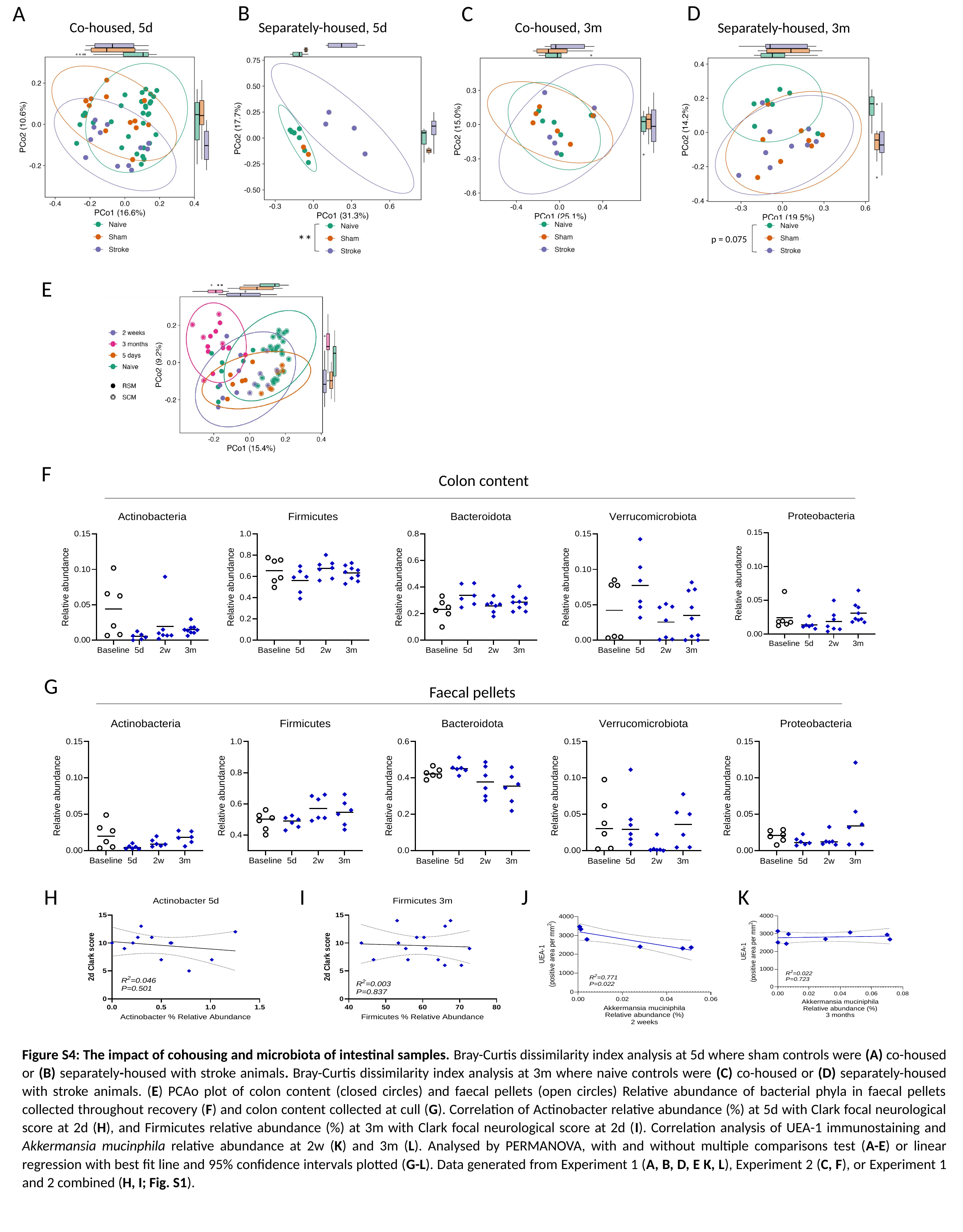

B
D
A
C
Co-housed, 5d
Co-housed, 3m
Separately-housed, 5d
Separately-housed, 3m
**
p = 0.075
E
F
Colon content
G
Faecal pellets
H
I
J
K
Figure S4: The impact of cohousing and microbiota of intestinal samples. Bray-Curtis dissimilarity index analysis at 5d where sham controls were (A) co-housed or (B) separately-housed with stroke animals. Bray-Curtis dissimilarity index analysis at 3m where naive controls were (C) co-housed or (D) separately-housed with stroke animals. (E) PCAo plot of colon content (closed circles) and faecal pellets (open circles) Relative abundance of bacterial phyla in faecal pellets collected throughout recovery (F) and colon content collected at cull (G). Correlation of Actinobacter relative abundance (%) at 5d with Clark focal neurological score at 2d (H), and Firmicutes relative abundance (%) at 3m with Clark focal neurological score at 2d (I). Correlation analysis of UEA-1 immunostaining and Akkermansia mucinphila relative abundance at 2w (K) and 3m (L). Analysed by PERMANOVA, with and without multiple comparisons test (A-E) or linear regression with best fit line and 95% confidence intervals plotted (G-L). Data generated from Experiment 1 (A, B, D, E K, L), Experiment 2 (C, F), or Experiment 1 and 2 combined (H, I; Fig. S1).

## Slide 3
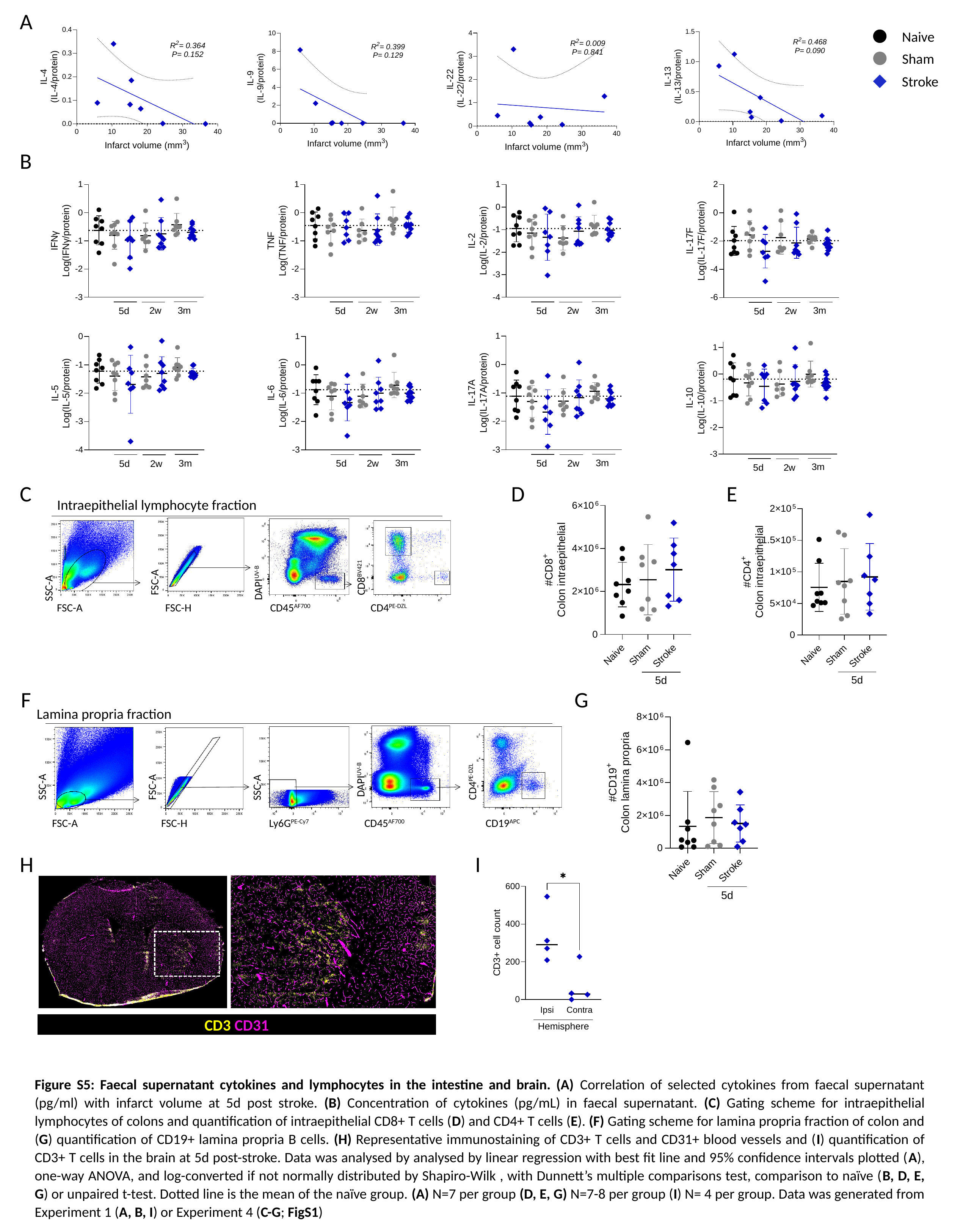

A
Naive
Sham
Stroke
B
C
D
E
Intraepithelial lymphocyte fraction
FSC-A
CD8BV421
DAPIUV-B
SSC-A
FSC-A
FSC-H
CD45AF700
CD4PE-DZL
F
G
Lamina propria fraction
DAPIUV-B
CD4PE-DZL
SSC-A
FSC-A
SSC-A
FSC-A
FSC-H
Ly6GPE-Cy7
CD45AF700
CD19APC
H
H
I
CD3 CD31
Figure S5: Faecal supernatant cytokines and lymphocytes in the intestine and brain. (A) Correlation of selected cytokines from faecal supernatant (pg/ml) with infarct volume at 5d post stroke. (B) Concentration of cytokines (pg/mL) in faecal supernatant. (C) Gating scheme for intraepithelial lymphocytes of colons and quantification of intraepithelial CD8+ T cells (D) and CD4+ T cells (E). (F) Gating scheme for lamina propria fraction of colon and (G) quantification of CD19+ lamina propria B cells. (H) Representative immunostaining of CD3+ T cells and CD31+ blood vessels and (I) quantification of CD3+ T cells in the brain at 5d post-stroke. Data was analysed by analysed by linear regression with best fit line and 95% confidence intervals plotted (A), one-way ANOVA, and log-converted if not normally distributed by Shapiro-Wilk , with Dunnett’s multiple comparisons test, comparison to naïve (B, D, E, G) or unpaired t-test. Dotted line is the mean of the naïve group. (A) N=7 per group (D, E, G) N=7-8 per group (I) N= 4 per group. Data was generated from Experiment 1 (A, B, I) or Experiment 4 (C-G; FigS1)

## Slide 4
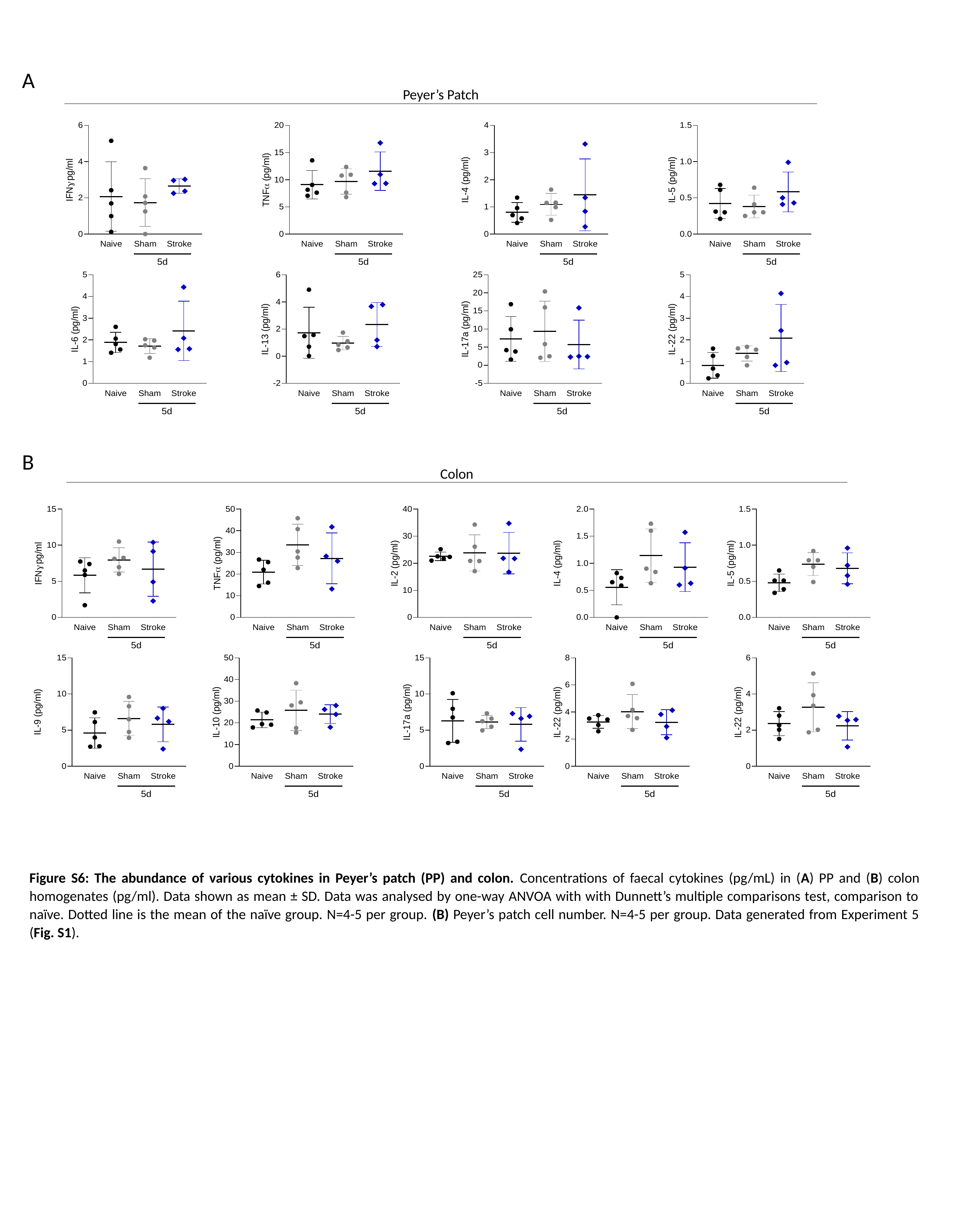

A
Peyer’s Patch
B
Colon
Figure S6: The abundance of various cytokines in Peyer’s patch (PP) and colon. Concentrations of faecal cytokines (pg/mL) in (A) PP and (B) colon homogenates (pg/ml). Data shown as mean ± SD. Data was analysed by one-way ANVOA with with Dunnett’s multiple comparisons test, comparison to naïve. Dotted line is the mean of the naïve group. N=4-5 per group. (B) Peyer’s patch cell number. N=4-5 per group. Data generated from Experiment 5 (Fig. S1).
